# A single-arm confirmatory trial of pazopanib in patients with paclitaxel-pretreated primary cutaneous angiosarcoma: Japan Clinical Oncology Group study (JCOG1605, JCOG-PCAS protocol)

**DOI:** 10.1186/s12885-020-07136-1

**Published:** 2020-07-13

**Authors:** Kohei Oashi, Taro Shibata, Kenjiro Namikawa, Akira Takahashi, Kenji Yokota, Eiji Nakano, Yukiko Teramoto, Arata Tsutsumida, Taku Maeda, Naoya Yamazaki, the Dermatologic Oncology Group of the Japan Clinical Oncology Group

**Affiliations:** 1 Department of Dermatology, Saitama Cancer Center, 780 Komuro, Ina, Kita-adachi-gun, Saitama, 362-0806 Japan; 2 JCOG Data Center/Operations Office, National Cancer Center Hospital, Tokyo, Japan; 3 Department of Dermatologic Oncology, National Cancer Center Hospital, 5-1-1 Tsukiji, Chuo-ku, Tokyo, 104-0045 Japan; 4 Department of Dermatology, Nagoya University Graduate School of Medicine, Nagoya, Japan; 5 Department of Skin Oncology/Dermatology, Saitama Medical University International Medical Center, Saitama, Japan; 6 Department of Dermatologic Oncology, Dermatology, Cancer Institute Hospital, Tokyo, Japan; 7 Department of Plastic and Reconstructive Surgery, Faculty of Medicine and Graduate School of Medicine Hokkaido University, Sapporo, Japan

**Keywords:** Angiosarcoma, Chemotherapy, Pazopanib, Single arm comfirmatory trial, Paclitaxel-resistant

## Abstract

**Background:**

Paclitaxel is a standard of care for patients with primary cutaneous angiosarcoma of the scalp and face. However, no standard second-line treatment for paclitaxel-resistant patients has ever been established. Since primary cutaneous angiosarcoma expresses a high level of vascular endothelial growth factor receptor, the multitargeted tyrosine kinase inhibitor pazopanib seemed to be the most promising agent, and several retrospective studies have demonstrated its activity against this disease. However, the efficacy and safety of pazopanib in paclitaxel-resistant patients with primary cutaneous angiosarcoma have never been evaluated in a clinical trial.

**Methods:**

In February 2018 the Dermatologic Oncology Group of Japan Clinical Oncology Group started a single-arm confirmatory trial to evaluate the efficacy and safety of pazopanib as a second-line treatment for patients with primary cutaneous angiosarcoma whose disease was resistant to paclitaxel or who were unable to tolerate paclitaxel (JCOG1605, JCOG-PCAS). Patients with primary cutaneous angiosarcoma not associated with lymphedema or radiation, progressing despite first-line paclitaxel monotherapy are included in the study. No prior systemic chemotherapy other than paclitaxel is permitted. Pazopanib is administered orally at an initial dosage of 800 mg once daily. Dose modifications for adverse events are made according to the dose reduction criteria described in the protocol. Treatment is continued until recurrence, disease progression, unacceptable toxic effects, patient refusal, or death. The primary endpoint is progression-free survival, secondary endpoints include overall survival, response rate, disease control rate, adverse events, and serious adverse events. We plan to recruit 30 participants in 5.5 years from 23 Japanese institutions. The follow-up period is set as 1 year after completion of accrual. The study protocol was approved by the Japan Clinical Oncology Group Protocol Review Committee in December 2017. Ethical approval for this study was granted by Ethics Committee of each institute.

**Discussion:**

If the primary endpoint is met, pazopanib will be regarded as a standard of care for paclitaxel-resistant patients for whom no standard second-line treatment is established.

**Trials registration:**

Registry number: UMIN000031438 [http://www.umin.ac.jp/ctr/index.htm]. Date of Registration: 23/Feb/2018. Date of First Participant Enrollment: 8/Mar/2018.

## Background

Angiosarcomas are extremely rare forms of sarcoma which originate from vascular endothelial cells. The number of patients accounts for 1 to 2% of all soft-tissue and visceral sarcomas [1–4]. Angiosarcomas can be classified into five subtypes, including cutaneous angiosarcoma not associated with lymphedema (primary cutaneous angiosarcoma), cutaneous angiosarcoma associated with lymphedema, radiation-induced cutaneous angiosarcoma, angiosarcoma of deep soft tissue, and angiosarcoma of parenchymal organs [5]. They are regarded as closely related tumors which share similar pathological features rather than a single entity. Primary cutaneous angiosarcoma is the most frequent subtype of angiosarcoma which accounts for about 28% of all angiosarcoma patients. Primary cutaneous angiosarcoma typically develops in the head and neck region, especially in the scalp and upper face of elderly men. Angiosarcoma of the head and neck region has a poor prognosis with reported 5 year survival rates of 11 to 53% [1, 3, 4, 6–10].

The standard of care for localized cutaneous angiosarcoma in Western countries is radical surgery combined with adjuvant radiation therapy (RT) [11, 12]. Since Japanese patients tend to present with larger primary tumors and a poorer prognosis in comparison with Caucasian patients [1, 5, 13–15], systemic chemotherapy (adjuvant and/or neoadjuvant) in combination with local treatment can be a viable option for Japanese patients [16].

Although doxorubicin has been a key drug in the treatment of metastatic and unresectable soft tissue sarcomas [17], since paclitaxel has been shown to be more effective against primary cutaneous angiosarcoma of the scalp and face [2, 18, 19], multimodal treatment, including local treatment and chemotherapy with paclitaxel has become a standard of care for Japanese patients with locoregional primary cutaneous angiosarcoma of the scalp and face. Because systemic chemotherapy with paclitaxel is also a first-line treatment for patients with distant metastasis, paclitaxel is administered to Japanese patients with primary cutaneous angiosarcoma as an initial treatment regardless of the extent of their disease. However, no standard second-line treatment for paclitaxel-resistant patients has ever been established, and the prognosis of such patients is dismal.

The recent development of molecular targeted therapy for cancer has resulted in the availability of many potential drugs for the treatment of primary cutaneous angiosarcoma [20–22]. In addition to molecular targeted therapies, several new cytotoxic agents, including eribulin and trabectedin, have shown to be effective in the treatment of advanced sarcoma [23, 24]. Unfortunately, these clinical trials have seldom or never included patients with primary cutaneous angiosarcoma, and thus a new clinical trial focusing on primary cutaneous angiosarcoma alone has been needed to evaluate the efficacy of these new drugs. Since primary cutaneous angiosarcoma expresses a high level of vascular endothelial growth factor receptor (VEGFR) [25, 26], the multitargeted tyrosine kinase inhibitor pazopanib, which blocks VEGFR- 1, 2, and 3, the platelet-derived growth factor receptor (PDGFR), and c-Kit, seemed to be the most promising agent [27], and several retrospective studies have demonstrated its activity against this disease [28–32]. However, the efficacy and safety of pazopanib in patients with primary cutaneous angiosarcoma have never been evaluated in a clinical trial.

In February 2018, the Dermatologic Oncology Group of Japan Clinical Oncology Group (JCOG) therefore started a single-arm confirmatory trial to evaluate the efficacy and safety of pazopanib as a second-line treatment for patients with primary cutaneous angiosarcoma whose disease was resistant to paclitaxel or who were unable to tolerate paclitaxel (JCOG1605, JCOG-PCAS).

## Methods / design

### Aim

The purpose of this study is to evaluate the efficacy and safety of pazopanib as a second-line treatment for patients with primary cutaneous angiosarcoma after failure of first-line paclitaxel.

### Study setting

A multi-institutional, single-arm, open-label, confirmatory trial.

### Funding

This study is supported in part by the National Cancer Center Research and Development Fund of Japan (2020-J-3).

### Endpoints

The primary endpoint is progression-free survival (PFS). PFS is defined as the time from registration to either the first event of tumor progression or death from any cause, and it is censored at the latest day when the patient is alive without any evidence of progression.

The secondary endpoints are overall survival (OS), response rate (RR), disease control rate (DCR), adverse events (AEs), and serious adverse events (SAEs). OS is defined as the time from registration to death from any cause, and it is censored at the last day the patient is known to be alive. RR is defined as the proportion of patients whose best overall response without confirmation is complete response (CR) or a partial response (PR) out of patients with measurable lesion at the baseline. Patient response is measured in accordance with the Response Evaluation Criteria in Solid Tumors, version 1.1 (RECIST 1.1). DCR is defined as the proportion of patients whose best overall response without confirmation is CR, PR, or stable disease (SD) out of patients with measurable lesion at the baseline. AEs are evaluated according to the Common Terminology Criteria for Adverse Events version 4.0 (CTCAE 4.0). SAE is defined as any ≧grade 4 non-hematologic toxicity at least possibly related to treatment, death from any cause during treatment or within 30 days after the last administration, and treatment-related death.

### Inclusion criteria

1. Histologically confirmed primary cutaneous angiosarcoma that is not associated with lymphedema or a history of radiation exposure.
2. Primary or metastatic lesions (histological evaluation is not essential for metastatic lesions).
3. Absence of intracranial metastasis.
4. Age between 20 years and 85 years old.
5. Eastern Cooperative Oncology Group (ECOG) performance status of 0 or 1.
6. Past history of paclitaxel monotherapy as a first-line treatment in combination with or not in combination with any local treatment (surgery and/or radiation therapy).
7. No past history of any systemic chemotherapy other than paclitaxel.
8. Resistance or intolerance to paclitaxel.
9. A measurable lesion is not required.
10. No prior use of antiangiogenic agents.
11. Absence of any non-healing wound.
12. Adequate organ and marrow function as defined below within 14 days prior to enrollment:

- Absolute neutrophil count ≧1500/mm^3^.
- Hemoglobin ≧9.0 g/dL.
- Platelet count ≧10 × 10^4^/mm^3^.
- Total bilirubin ≦2.25 mg/dL.
- Aspartate aminotransferase ≦75 U/L.
- Alanine aminotransferase, male: ≦105 U/L, female: ≦57.5 U/L.
- Renal function: serum creatinine ≦1.5 mg/dL, or, if > 1.5 mg/dL, calculated creatinine clearance > 50 mL/min.
- Prothrombin time-international normalized ratio ≦1.38.
- Activated partial thromboplastin time ≦44.4 s.
- Thyroid-stimulating hormone: 0.5–4.5 μU/mL.
- Free triiodothyronine: 2.0–4.0 pg/mL.
- Free thyroxine: 0.9–1.8 ng/dL.
- Left ventricular ejection fraction ≧50%.
- Corrected QT interval ≦480 ms.
- Negative urinary protein, or, if not negative, 24-h urinary protein excretion ≦0.15 g (urinary protein/creatinine ratio ≦0.15 g/gCr is permitted).

13) Written informed consent.

### Exclusion criteria

1. A synchronous or metachronous (within 3 years) malignancy except for carcinoma in situ or intramucosal tumors curatively treated by local therapy with a 5-year survival of over 95%.
2. Active infection requiring systemic therapy.
3. Body temperature ≧38 degrees Celsius.
4. Pregnant, possibly pregnant, or lactating women. Within 28 days after childbirth. Men favoring gestation of their partners.
5. Severe psychiatric disease.
6. Current systemic administration of a steroid or other immunosuppressant.
7. Hypertension (> 140 mmHg systolic and > 90 mmHg diastolic) that cannot be adequately controlled with antihypertensive treatments.
8. Any cardiovascular disease or procedure listed below within 6 months prior to enrollment:

- Percutaneous transluminal coronary angioplasty.
- Myocardial infarction.
- Unstable angina pectoris.
- Coronary artery bypass grafting.
- Symptomatic peripheral arterial disease.
- New York Heart Association class III or IV heart failure.

9) Massive pleural or pericardial effusion.
10) Interstitial lung disease.
11) Intestinal paralysis or ileus.
12) Active bleeding.
13) Cerebrovascular event within 6 months prior to enrollment.
14) Pulmonary embolism within 6 months prior to enrollment.
15) Untreated deep vein thrombosis within 6 months prior to enrollment.

### Treatment

Pazopanib is administered orally at an initial dosage of 800 mg once daily. Dose modifications for adverse events including hypertension, hepatic disorders, and cardiac dysfunction are made in accordance with the dose reduction criteria. Treatment is continued until recurrence, disease progression (according to RECIST version 1.1), unacceptable toxic effects, and withdrawal of consent or death.

### Follow-up

All patients will be followed up for at least 1 year after patient accrual is completed. Enhanced cervical, thoracic, abdominal and pelvic computed tomography (CT) images will be evaluated at least every 4 weeks until disease progression or death. Tumor response will also be assessed every 4 weeks according to RECIST version 1.1. Physical examination, laboratory tests, and evaluation of adverse events according to CTCAE ver. 4.0 will be carried out at least every 2 weeks during protocol treatment.

### Statistical analysis

This study is designed as a single-arm, confirmatory study to confirm the efficacy and safety of pazopanib as a second-line treatment in patients with primary cutaneous angiosarcoma that has become resistant or in patients who have become intolerant to paclitaxel. The planned sample size is 30, which will provide 70% power under the hypothesis of the median PFS being the expected value of 5.0 months and a threshold value of 3.0 months using a one-sided alpha of 0.1.

### Interim analysis and monitoring

We plan to conduct futility analyses to judge whether the study should be terminated early due to futility. The futility analyses will be conducted after half of the planned number patients have been enrolled. If the upper limit of the 95% confidence interval (CI) of the median PFS is less than the expected value, a decision on the study termination will be made via a comprehensive review at that time. The JCOG Data Center staff and the Study Coordinator will conduct central monitoring issuing a monitoring report at least every 1 year to evaluate and improve study progress, data integrity and patient safety. Futility will be considered at each monitoring report if necessary. For quality assurance, site-visit audits, not for a study-specific basis but for the study group basis, will be performed by the JCOG Audit Committee.

## Discussion

The aim of this study is to evaluate the efficacy and safety of pazopanib as a second-line treatment for patients with primary cutaneous angiosarcoma after failure of first-line paclitaxel. If the primary endpoint is met, pazopanib will be regarded as a standard of care for paclitaxel-resistant patients for whom no standard second-line treatment is established. This study is designed as a single arm trial considering rarity of disease. Given the fact that expected and threshold value of primary endpoint was set according to robust historical control data (unpublished), we position this study as a confirmatory trial.

## Data Availability

Data sharing is not applicable to this article as no datasets were generated or analysed at the time of submission.

## Abbreviations

RT: Radiation therapy
VEGFR: Vascular endothelial growth factor receptor
PDGFR: Platelet-derived growth factor receptor
JCOG: Japan Clinical Oncology Group
PFS: Progression-free survival
OS: Overall survival
RR: Response rate
DCR: Disease control rate
AEs: Adverse events
SAEs: Serious adverse events
CR: Complete response
PR: Partial response
RECIST: Response Evaluation Criteria in Solid Tumors
CTCAE: Common Terminology Criteria for Adverse Events
ECOG: Eastern Cooperative Oncology Group
JCOG: The Japan Clinical Oncology Group
CI: Confidence interval
CT: Computed tomography
